# Comparative evaluation of apical debris extrusion during root canal preparation using three different rotary file systems

**DOI:** 10.12688/f1000research.135235.2

**Published:** 2023-10-06

**Authors:** Rutuja Rajnekar, Nikhil Mankar, Pradnya Nikhade, Manoj Chandak

**Affiliations:** 1 Datta Meghe Institute of Higher Education and Research, Sawangi, Maharashtra, India; 2 Department of Conservative Dentistry and Endodontics, Sharad Pawar Dental College, Wardha, Maharshtra, 442001, India

**Keywords:** Apical extrusion, TruNatomy, ProTaper Next, 2Shape

## Abstract

**Background**: Endodontic success depends on the complete cleaning and shaping of the root canal. In order to achieve this goal, debris removal is essential. Even with improvements in instrument design, apical debris extrusion continues to be a cause of periradicular inflammation. The current study aimed to compare the amount of apically extruded debris throughout the canal instrumentation with TruNatomy, ProTaper Next, 2Shape rotary systems.

**Methods**: A total of 60 freshly extracted single‑rooted mandibular premolars were used. An access opening was made, and a working length was estimated. The samples were arbitrarily allocated into three groups *i.e.* Group I: TruNatomy (n = 20), Group II: ProTaper Next (n = 20), Group III: 2Shape (n = 20). During the canal instrumentation, the extruded debris were collected in preweighed Eppendorf tubes. Post-instrumentation, the tubes were placed in a hot air oven at 140°C for five hours. For calculating the dry debris weight, the tube’s pre-instrumentation weight was deducted from the post-instrumentation weight. The data was analysed using one‑way analysis of variance and *post hoc* Tukey’s test.

**Results**: Extrusion of debris was noted in all the specimens. TruNatomy rotary system showed least debris extrusion in comparison to ProTaper Next and 2Shape (P < 0.05). However, the results were statistically non-significant between ProTaper Next and 2Shape (P > 0.05).

**Conclusions**: It was noted that all instruments apically extruded debris, with TruNatomy system being related to minimum extrusion.

## Introduction

The field of endodontics has experienced a revolution in recent years, with treatment becoming more precise and prognosis becoming more predictable. Endodontic therapy necessitates meticulous biomechanical preparation, three-dimensional obturation, and a leakage-free coronal restoration for its success. ^1^ In addition to removing all irritants from the canal, the instrumentation should also facilitate debridement without injuring periapical tissues. ^2^ Despite efforts to maintain the correct working length throughout the root canal instrumentation, debris such as pulpal fragments, microbes, necrotic remnants, dentinal chips, and irrigants are forced beyond the apex in the periradicular tissues. ^3^

Nevertheless, this debris extrusion can induce an inflammatory response which may compromise healing, cause flare up, or lead to failure in the long/short term. Patients with flare-up experience pain, swelling, or both during and after their endodontic treatment, leading to emergency appointments. ^4^ Therefore, preventing debris extrusion is important during root canal procedures. Chapman *et al.* reported for the first time that infective material was extruded periapically during root canal instrumentation. ^5^ Vande Visse and Brilliant calculated the apical debris extrusion during the biomechanical preparation for the first time. ^6^ As a result of Myers and Montgomery’s research, an approach for evaluating the amount of extruded debris was developed. ^7^ Tanalp and Gungor stated that the apical extrusion of bacterial products, pulpal tissue, and irrigants are one of the reasons for endodontic failure. ^8^

It has been observed that debris extrusion varies between different instrumentation systems. It is believed that this is because of variations in cutting blade and cross-sectional design, tip type, taper, configuration, the number of files used, flexibility, alloy, kinematics, and cutting efficiency. ^9^

Many authors believe that the extrusion of some debris is unavoidable during instrumentation, and that there is no method for completely eliminating this. Nevertheless, some instrumentation system may extrude fewer debris compared to others.

The TruNatomy (Dentsply Sirona, Maillefer, Ballaigues, Switzerland) rotary file system is fabricated from 0.8 mm NiTi wire in contrast to the standard files, which are manufactured from 1.2 mm NiTi wire. ^10^ The system includes an orifice opener; glider; three shaping files which are Small (20/4%); Prime (26/4%); and Medium (36/3%). These files have an off-centered parallelogram cross-section which provides extra space for debridement. ^11^

The ProTaper Next (Dentsply Maillefer, Ballaigues, Switzerland) rotary system is produced from M-Wire technology. ^12^ These files have an asymmetrical rectangular cross-sectional design with variable taper. This results in snake-like swaggering motion along its active portion, decreasing the screw-in effect and taper-lock. ^13^ The offset design of this system causes maximum amount of debris to be forced out of the canal. ProTaper Next system includes X1 (17/4%), X2 (25/6%), X3 (30/7%), X4 (40/6%) and X5 (50/6%) rotary files.

2Shape (MicroMega, Besancon, France) rotary files are made of T-wire alloy having off-centered triple helix cross-sectional design. The file design has two cutting edges, which enhances the cutting efficacy with a secondary cutting edge intended for better debris removal. 2Shape consists of two shaping instruments and an optional file for apical finishing. The 2Shape system consist of TS1 (25/4%), TS2 (25/6%), F35 (35/6%), and F40 (40/4%) rotary files. ^14^

As discussed the apical debris extrusion are one of the reason for flareups, therefore preventing debris extrusion is necessary. This study aimed to estimate the amount of debris extrusion during instrumentation of root canals by TruNatomy, ProTaper Next and 2Shape rotary systems, and evaluate the effectiveness of their design in minimizing the amount of apical debris extrusion during use with the Myers and Montgomery’s method. The null hypothesis considered was that there was no difference in the amount of apical debris extrusion between the rotary systems.

## Methods

### Study design

After obtaining clearance from the Institutional Research Ethics Committee of Datta Meghe Institute of Higher Education and Research, the ethical approval letter was obtained (Ref. No. DMIMS (DU)/IEC/2020-21/9388). This was an *in vitro* study design.

### Sample size calculation

The formula used for sample size calculation based on previous studies was:

$$n=\left(\mathrm{Z\alpha}+\mathrm{Z\beta}\right)2\left(\delta_{1}2+\delta_{2}2/K\right)/∆2$$

Where Z _α_ = level of significance at 5% *i.e.*, 95% confidence interval = 1.96

Z _β_ = power of test = 80% = 0.84

δ1 = SD of mean debris in Group 1 = 0.00024

δ2 = SD of mean debris in Group 2 = 0.00018

∆=Difference between two groups=0.00065−0.00045=0.0002

K = 1

$$n={\left(1.96+0.84\right)}^{2}\left(0.00024^{2}+0.00018^{2}/1\right)/0.0002^{2}=17.49=20\;\text{Samples needed in each group}$$

### Data collection

*Preparation of samples*

Sixty freshly extracted single-rooted mandibular premolars having less than 10° curvature were included in the experimental study. All the samples were inspected under a Dental Operating Microscope for confirmation of completely formed root apices. The digital radiographs were taken in buccal and proximal directions to confirm the existence of a single canal, complex anatomy, calcifications, and mature apex formation. All sample’s outer surface was cleaned for debris or remnants, which were further stored in normal saline. To standardize, all the samples were sectioned using diamond disc with 16 ± 0.20 mm tooth length.

A standard access opening was done using a high-speed diamond bur. Each canal was carefully inserted with a #10 K File (Mani Inc, Japan) until it could be barely seen at the apex. Further, the file stopper was adjusted at the occlusal surface, which was used as the reference point. From this measured length, 1 mm was subtracted for each sample’s working length determination. The teeth in which #10 K file was just visible at the apex along with the #15 K file, which snugly fits at the working length of the tooth, were included in the study.

*Instrumentation and debris collection*

**Figure 1.**
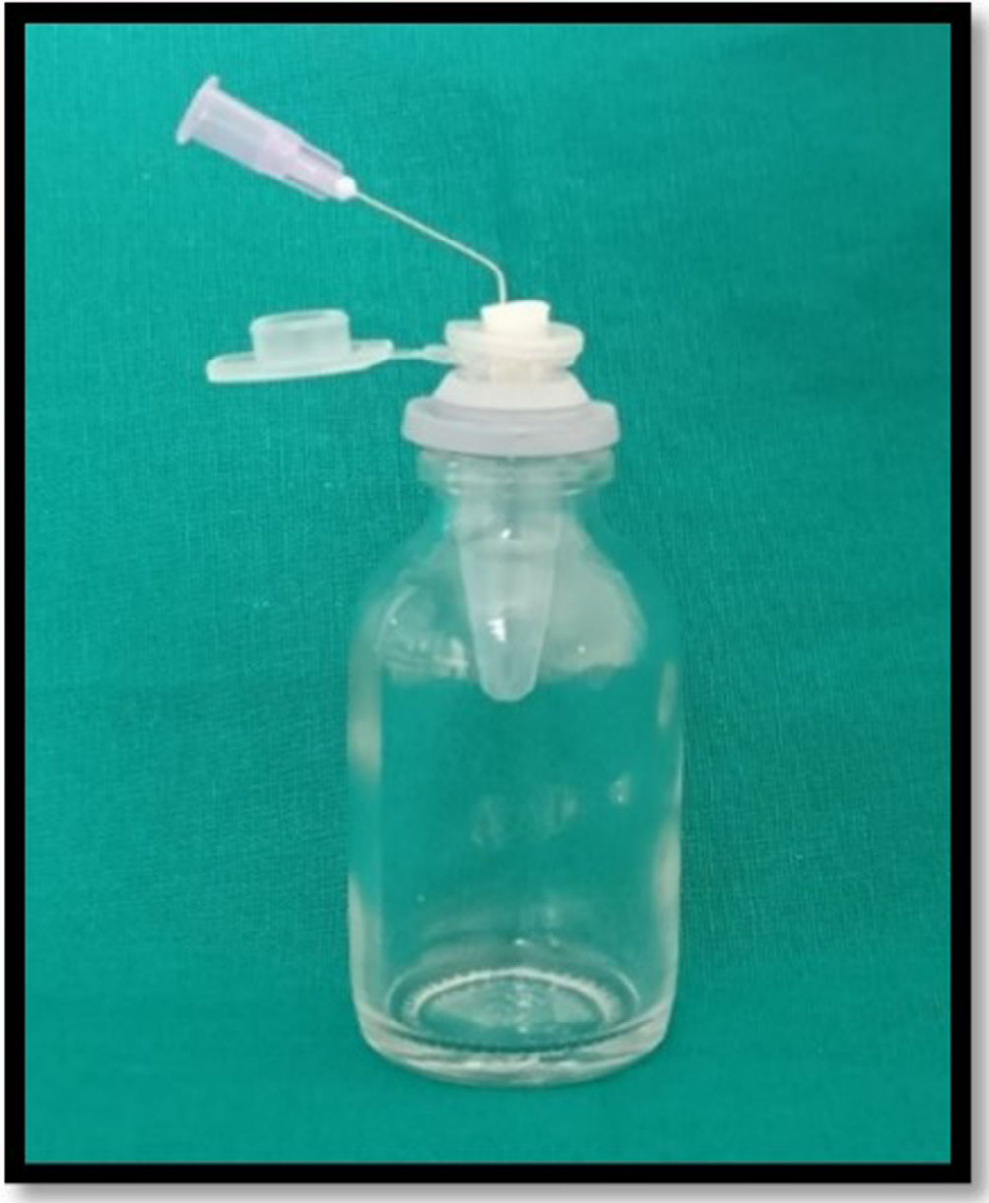
Debris collection apparatus according to Myers and Montgomery method.

**Figure 2.**
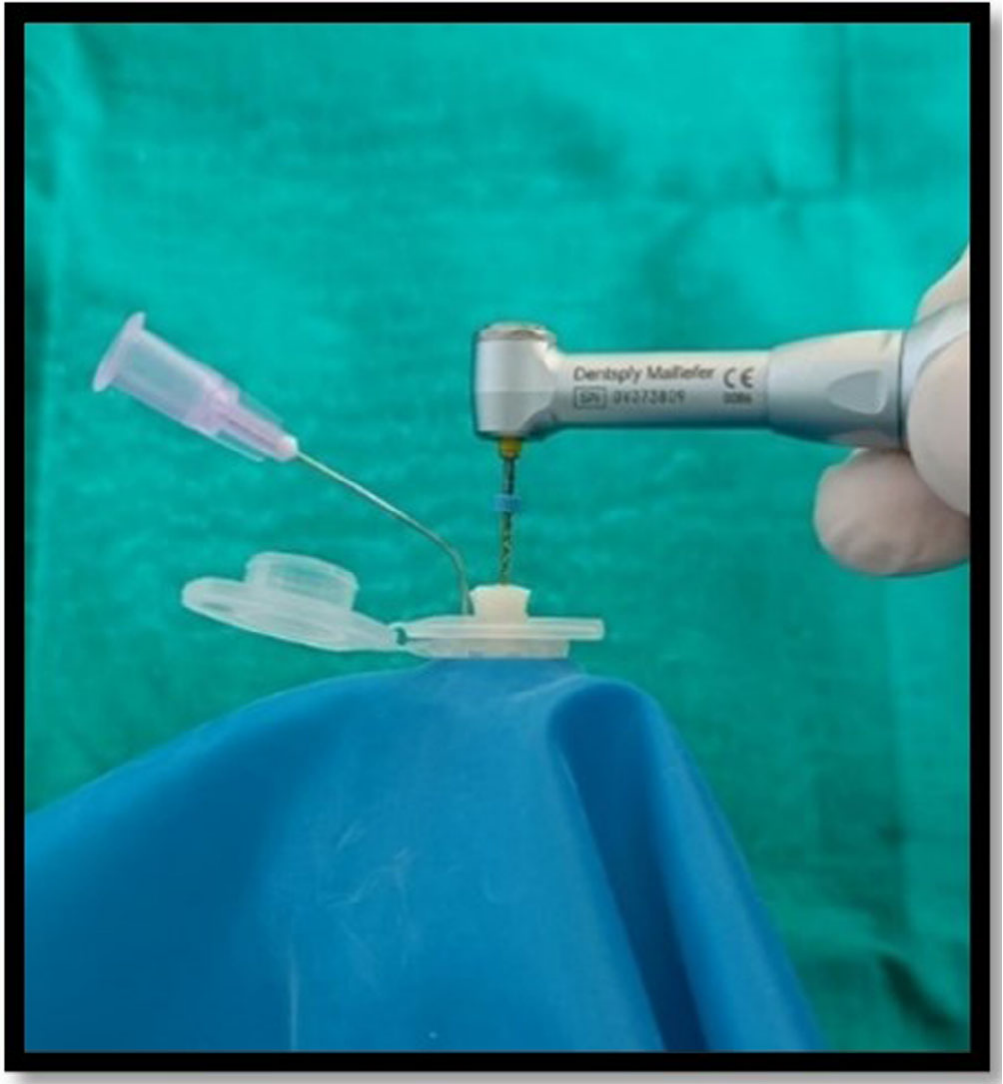
Biomechanical preparation of the samples in Debris collection apparatus.

The debris collection apparatus was set up in accordance with Myers and Montgomery’s study 1991 ^7^ ( Figure 1). For collecting the apically extruded debris throughout instrumentation, the Eppendorf tubes were used. A total of 60 tubes were preweighed on an analytical electronic microbalance of 10 ^-6^ g precision (Sartorius weighing Technology GmbH, Goettingen, Germany). Each tube’s weight was calculated by taking the mean of three consecutive measurements and placed into an empty glass vial. The teeth were forced into an Eppendorf tube after the preparation of the standard access cavity. Each Eppendorf tube was fitted in the empty vial through the rubber stopper. The apical portion of the samples were suspended in the Eppendorf tube. For equalization of the air pressure inside and outside the tubes, the tubes were vented by inserting a 24-gauge needle along the side of the stopper. When the canal instrumentations were performed, the vials were coated with a dental dam sheet so as to prevent the operator’s view of the tooth’s apical portion ( Figure 2). In order to assess debris extrusion, another examiner was blinded regarding different groups.

*Experimental groups*

All the samples were randomly distributed into three experimental groups on the basis of instrumentation techniques used.

Group I: TruNatomy

As per the manufacturer’s instructions, a smooth glide path with the #10 K or #15 K File was obtained. TruNatomy Orifice modifier was used till half of the working length, followed by glide path preparation by the TruNatomy Glider. The shaping file used was TruNatomy Prime (26/4%), at 1.5 torque and 500 rpm till the working length.

Group II: ProTaper Next

Following the manufacturer’s recommendations, the ProTaper Next rotary system was used with pecking motion. The Glide path was established with #15 K File. The X1 (17/4%) file, followed by the X2 (25/6%) file, was used with in-and-out pecking action till the working length at 2 N/cm torque and 300 rpm, according to manufacturing company recommendations.

Group III: 2Shape

After achieving a glide path with #10 K or #15 K File, 2Shape rotary files were used with pecking motion. The TS1 file (25/4%) was used with pecking motion followed by shaping with the TS2 file (25/6%) at 1.2 N/cm torque and a speed of 300 rpm till working length was achieved.

Irrespective of the instrumentation system, 1 mL distilled water with a 30-gauge side vented needle was used for irrigating the canals following each instrument. Total irrigant used was 8 mL.

Evaluation of apically extruded debris

**Figure 3.**
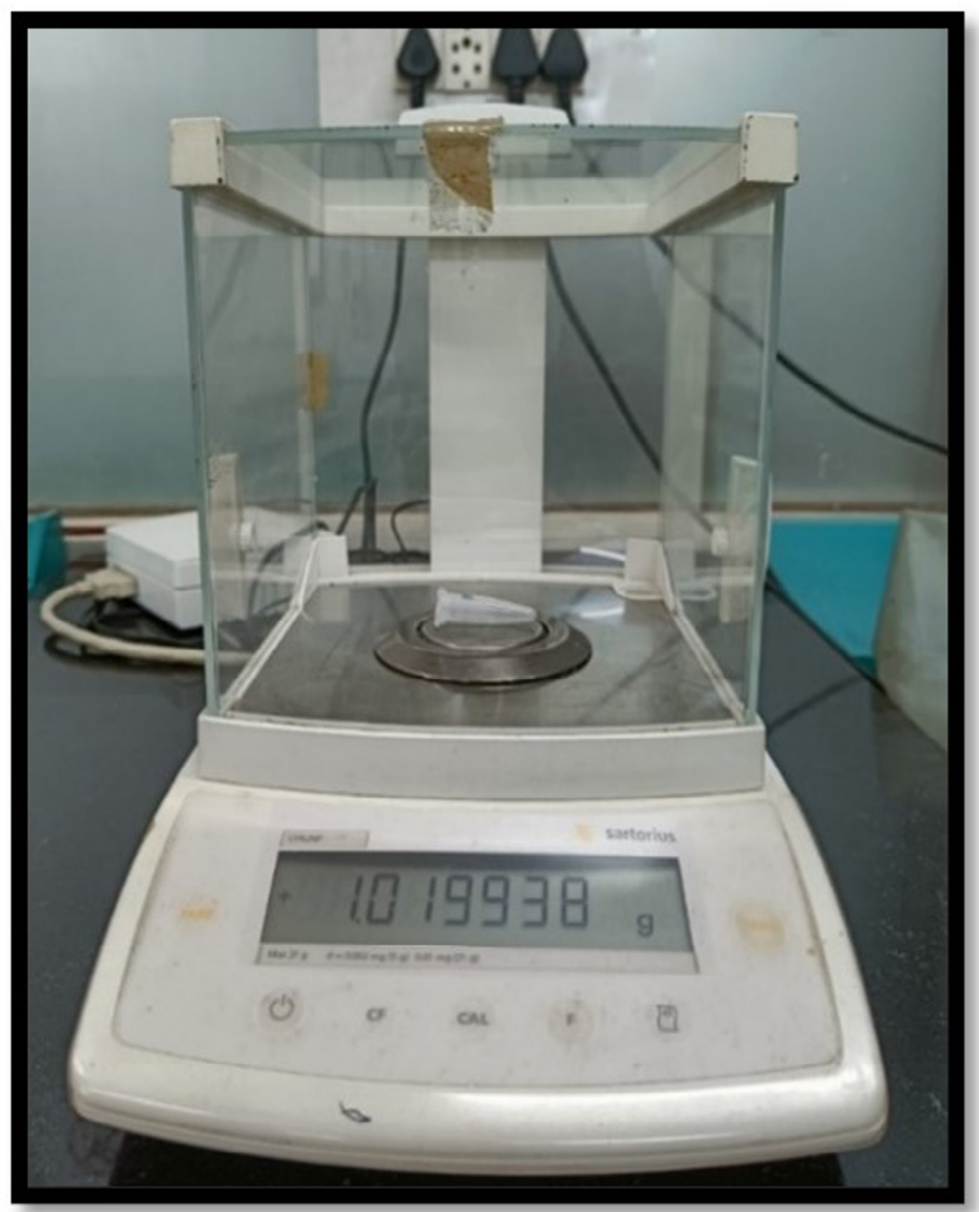
Electronic microbalance with 10 ^-6^ g precision.

Post-instrumentation, each sample was removed from the Eppendorf tube. To collect the root surface-adhered debris, the root’s apical portion was washed with 1 mL distilled water in a tube. Then, these Eppendorf tubes having debris, extruded irrigants and distilled water were locked with the lid. The tubes were further stored at 140° in a Hot Air Oven for five hours. This led to evaporation of the moisture, obtaining dry debris. An electronic microbalance (10 ^-6^ g precision) was used to weight the dry debris and was repeated three times by the same operator, and the average reading was noted ( Figure 3). For the assessment of final weight of dry extruded debris in each tube, the mean pre-instrumentation weight of the tube was deducted from their mean post-instrumentation weight. All three groups were compared. The results were statistically analysed.

### Data analysis

The obtained data was statistically analysed using ANOVA and *post hoc* Tukey test using SPSS version 21 (SPSS Inc., Chicago, IL, USA). The amount of extruded debris was statistically analyzed, and the level of significance was set at P < 0.05.

## Results

In order to analyze the data, mean and standard deviation were calculated ( Table 1) and subsequently subjected to ANOVA. Each instrumentation technique had a significant difference in debris extrusion. In order to compare the groups, *post hoc* Tukey’s tests were used in which TruNatomy showed a significant reduction in debris extrusion when compared with ProTaper Next and 2Shape (P < 0.05). When compared between ProTaper Next and 2Shape, there was similar debris extrusion with ProTaper Next and 2Shape, and the difference was statistically non-significant (P > 0.05) ( Table 2).

**Table 1.** Mean values and standard deviations of the amount of extruded debris in the experimental groups.

Groups	Mean	Standard deviations
TruNatomy	0.0003	0.0001
ProTaper Next	0.0009	0.0001
2Shape	0.0009	0.0001

**Table 2.** Intergroup comparison between weight of debris using *post hoc* Tukey’s Test.

Groups	Difference	p-value
**TruNatomy *versus* ProTaper Next**	0.0006 ± 0.0002	0.001 *
**TruNatomy *versus* 2Shape**	0.0006 ± 0.0002	0.001 *
**ProTaper Next *versus* 2Shape**	0.00005 ± 0.0002	0.73

* p-value < 0.05 is statistically significant.

## Discussion

The purpose of root canal therapy is to remove the dentinal debris, necrotic pulp tissues, and microorganisms, leaving the root canals clean and shaped. ^15^ One of the biological objective of Schilder’s cleaning and shaping is prevention of apical extrusion of debris and irrigants into the periradicular tissues. The apical extrusion of infected debris may pose a clinically significant complication since it may lead to the postoperative pain or extraradicular infection.

The present study created standard conditions (other than the instrumentation system used) for all groups. For standardization of tooth morphology in the current research, 60 single-rooted mandibular premolars were selected based on the canal size, curvature and working length. To avoid any potential variation in debris extrusion caused by each sample’s different working length, all the sample teeth were decoronated before instrumentation.

The current study ensured equal distribution between the experimental groups by standardizing the size of apical foramen. As a result, the sample was excluded if a #15 K file was found to extend beyond the apex. In the current study, sodium hypochlorite was not used for irrigation as suggested by Tanalp and Gungor in 2014 to prevent weight bias caused by the crystallization of sodium hypochlorite, which cannot be distinguished from the weight of actual extruded debris. ^8^ Irrigation was strictly restricted to 8 mL of distilled water, which leaves no residue once evaporated.

The various engine-driven NiTi file systems available in the market today have different cross-sectional designs, all of which are associated with some amount of debris extrusion. Since Trunatomy rotary files are newly introduced, they are used to compare with other file system. All the rotary files used in the study are 5 ^th^ generation rotary file systems i.e., offset cross sectional design for effective debridement. Hence, the present study was performed to analyze and compare the amount of extrusion of debris associated with TruNatomy; ProTaper Next; and 2Shape file systems.

As compared to all the instruments tested in this study, the least amount of debris extrusion was associated with TruNatomy system (Group I) followed by 2Shape (Group III) and ProTaper Next (Group II). There were statistically significant differences between TruNatomy versus ProTaper Next (p-value = 0.001); and TruNatomy versus 2Shape (p-value = 0.001), whereas no statistically significant difference was found between 2Shape and ProTaper Next.

The minimum amount of debris produced by TruNatomy files may be due to the off-centered parallelogram cross-section, leading to less contact between the file and the dentin, *i.e.* the file touched the dentin only at two points at a time, providing additional space for coronal debris extrusion. ^16^ Few studies stated an association between the instrument taper and the amount of debris extrusion. A larger apical taper could lead to more aggressive preparation of the canals, which may cause more apical debris extrusion. ^17^ The results of the present study supported this finding, since TruNatomy has a 4% taper, whereas 2Shape and ProTaper Next had a taper of 6% at the tip. Moreover, contradictory to this, earlier findings stated that the larger taper did not essentially cause larger amount of debris to be extruded apically. ^18^ The current study findings are in accordance with Mustafa *et al*. and Cirakoglu, Ozbay. ^11^^,^^13^ They concluded that TruNatomy instruments are associated with significantly lesser debris extrusion than ProTaper Next. Contradictory to above findings, T Al Omari et al stated VDW Rotate system extruded less debris compared to trunatomy files. ^19^

The features of instrument systems, like cross-sectional design, kinematics, tip diameter, and instrument taper, all affect debris extrusion. ^9^ 2Shape files have a cross-sectional design of an asymmetric triple helix *i.e.* it causes a non-uniform, lesser contact points between the file and the root dentin. ^14^ These files have two leading cutting edges for enhanced cutting efficiency along with one secondary cutting edge for enhanced debris removal. In a study by Jamil and Ibraheem, they found more debris extrusion with 2Shape compared to Trunatomy system. ^20^

The ProTaper Next has an off-centered rectangular cross-sectional design. This design leads to only two-point contact with the canal wall at a time. It works in a snake-like swaggering motion leading to removal of more debris out of the canal. ^21^ However, there may be a significant amount of debris pushed out of the apical third due to a greater taper on the instruments at the apical 3 mm.

In the current study, debris extrusion for both the groups *i.e.*, 2Shape and ProTaper Next, had similar amount of extrusion. However, Paradkar *et al*. found less debris extrusion with 2Shape compared to ProTaper Next system. ^22^ Similar findings were stated by Ghoneim, Shaheen and Alani, Al-Huwaizi. ^14^^,^^23^

Furthermore, several studies found that more extruded debris could be attributed to a greater number of files used during preparation. ^24^^,^^25^ Hence, when more files are required to achieve the appropriate apical foramen size during instrumentation, it could lead to more significant debris extrusion. ^26^ This could contradict our results, as TruNatomy instruments caused significantly less debris extrusion than the ProTaper Next and 2Shape. The TruNatomy comprises of three files *i.e.*, an Orifice opener, a Glider, and the Prime file, whereas both 2Shape and ProTaper Next systems are comprised of only two files. However, Nevares *et al*. and Bilgi *et al*. ^27^^,^^28^ stated the number of instruments did not seem to influence the results. Another factor for the least debris extrusion in TruNatomy could be coronal flaring, since the system consists of an orifice modifier. Leeb stated coronal enlargement of an orifice cause early access to irrigating solutions. ^29^ It allows shaping files to prepare the apical portion with reduced contact with root dentin and, therefore, less friction. Topcuoglu *et al*. in their study mentioned that there is less apical debris extrusion when coronal flaring is done because the quantity of dentin available to extrude apically is minimized here, and it also provides a larger space for the debris to be washed out in a coronal direction when it is produced. ^30^

In the present study, all the systems used were in continuous rotation, so there was no variable regarding instrument kinematics. A systematic review by Caviedes-Bucheli *et al*. stated that the rotary instrument’s cross-sectional design significantly affected the quantity of debris extruded more than the motion kinematics. ^31^

As per the result of the study, when all the conditions were standardized, all systems caused debris to be extruded apically. There are no methods that ultimately prevent debris extrusion, and the findings of the present study are consistent with those of previous studies.

Tanalp *et al*. suggested that despite the absence of the specific threshold value of irritation, a smaller quantity of extruded material may be more prone to initiate a periradicular reaction if it is associated with a high bacterial content exhibiting high antigenic as well as virulence characteristics contrary to the high amount of extruded debris. ^8^

Therefore, the operator must make every effort to minimize the debris extrusion during instrumentation, which is in the operator’s hands to a large extent.

This study had limitations: the experimental model can’t reflect a clinical situation since periradicular tissues and bone naturally resist debris and irritant distribution. The apically extruded debris were measured and compared in single-rooted teeth with a single canal; the results may vary for multiple canals in single- or multi-rooted teeth. Sample selection was limited to teeth having a mature apex. The outcome may not match with the teeth showing open apices.

Therefore, it was concluded that all three instrumentation systems led to debris extrusion even if the working length was kept 1 mm short of the root apex. Since this was an experimental study, due to the lack of periapical tissues providing the back pressure, debris extrusion could have varied. Within these limitations, the TruNatomy extruded a significantly lesser amount of debris compared to ProTaper Next and 2Shape system.

## Data Availability

Zenodo: Comparative evaluation of apical debris extrusion during root canal preparation using three different rotary file systems, https://doi.org/10.5281/zenodo.7940511. ^32^ This project contains the following underlying data:
- Book1.csv Zenodo: STROBE checklist for “Comparative evaluation of apical debris extrusion during root canal preparation using three different rotary file systems”, https://doi.org/10.5281/zenodo.7940628. ^33^
