# Supplementary material for: Comparative evaluation of apical debris extrusion during root canal preparation using three different rotary file systems
Source: F1000Res. 2023 Oct 6;12:797. Originally published 2023 Jul 10. [Version 2] doi: 10.12688/f1000research.135235.2 (PMC11362740; doi:10.12688/f1000research.135235.2)
Supplement: Peer review history

# Reviewer report: Reviewer response for version 2

**DOI:** 10.5256/f1000research.156987.r302251

**Published:** 2024-09-19

**Authors:** Balaji Suresh

**Affiliations:** 1 Saveetha Institute Of Medical and Technical Sciences, Chennai, India

Short Summary of the Article

 The article investigates the amount of apical debris extrusion during root canal instrumentation using three different rotary file systems: TruNatomy, ProTaper Next, and 2Shape. The study's goal was to determine which system minimizes debris extrusion, a factor that can lead to postoperative complications like inflammation or treatment failure. The experimental design involved using 60 mandibular premolars, and debris was collected and measured using the Myers and Montgomery method. The study concluded that the TruNatomy system resulted in significantly less debris extrusion compared to ProTaper Next and 2Shape. However, both ProTaper Next and 2Shape exhibited similar levels of debris extrusion.

 Strengths of the Article

1. **Scientific Rigor**: The study is well-designed, with clear ethical approval and methodological rigor. The use of a controlled in vitro setting, with standardization of sample size, morphology, and working length, strengthens the validity of the findings. The experimental apparatus was set up in accordance with an established methodology (Myers and Montgomery), ensuring reliability.
2. **Comparative Analysis**: The article provides a detailed comparison of three widely-used rotary file systems. By focusing on TruNatomy, ProTaper Next, and 2Shape, the authors address a critical gap in knowledge about the relative efficiency of these systems in minimizing debris extrusion.
3. **Detailed Discussion**: The discussion thoroughly explains the factors influencing debris extrusion, such as file design, taper, cross-sectional configuration, and cutting efficiency. The authors also link their findings to previous studies, adding depth and credibility to their interpretations.
4. **Clinical Relevance**: The findings have practical implications for endodontic therapy, as minimizing apical debris extrusion is critical to avoiding postoperative pain, inflammation, or treatment failure. The study gives clinicians a clear indication that the TruNatomy system may offer better outcomes in terms of reducing complications related to debris extrusion.

Weaknesses of the Article

1. **Limited Clinical Applicability**: One major limitation of this study is that it was conducted in vitro, which does not fully mimic real clinical conditions. As the authors mention, the absence of periradicular tissues and bone in the experimental model means that the results may not entirely reflect what would happen in vivo, where these tissues would naturally resist debris extrusion.
2. **Single-Rooted Teeth Focus**: The study was restricted to single-rooted mandibular premolars with single canals, meaning that the results may not be generalizable to teeth with multiple roots or complex canal systems. The impact of these systems on multi-rooted teeth, which are common in clinical practice, was not explored.
3. **Limited Irrigation Protocol**: The study's irrigation protocol involved only distilled water, which, although justified to avoid the crystallization of sodium hypochlorite, does not reflect the clinical use of antimicrobial irrigants like sodium hypochlorite or EDTA, which might affect debris extrusion differently.
4. **Small Sample Size**: While the sample size calculation was done scientifically, 20 samples per group could be considered relatively small, especially when the goal is to compare the subtle differences in debris extrusion among instrumentation systems.

Additional Commentary

 The study effectively highlights the key differences between the three rotary file systems, particularly emphasizing how the unique design features of the TruNatomy system contribute to its reduced debris extrusion. The use of a smaller NiTi wire (0.8 mm) and the off-centered parallelogram cross-sectional design, which allows extra space for debris removal, were likely pivotal in reducing the contact area between the file and the canal wall, thus minimizing extrusion.

 However, while the findings suggest that TruNatomy is more favorable, the clinical impact of the differences in debris extrusion is not entirely clear. For example, while TruNatomy extruded less debris, it would be beneficial for future studies to assess whether this reduction leads to a significant decrease in clinical flare-ups, postoperative pain, or overall treatment success.

 Furthermore, expanding the study to include multi-rooted teeth and different canal morphologies would provide a more comprehensive understanding of how these systems perform in diverse clinical scenarios. Additionally, a more varied irrigation protocol could be tested to see if the same trends in debris extrusion persist when using clinically relevant irrigants.

 In conclusion, while the article presents valuable data on the effectiveness of different rotary file systems in reducing debris extrusion, there is room for further exploration to confirm these findings in clinical settings.

Is the work clearly and accurately presented and does it cite the current literature?

Yes

If applicable, is the statistical analysis and its interpretation appropriate?

I cannot comment. A qualified statistician is required.

Are all the source data underlying the results available to ensure full reproducibility?

Yes

Is the study design appropriate and is the work technically sound?

Yes

Are the conclusions drawn adequately supported by the results?

Yes

Are sufficient details of methods and analysis provided to allow replication by others?

Yes

Reviewer Expertise:

Pediatric dentistry

I confirm that I have read this submission and believe that I have an appropriate level of expertise to confirm that it is of an acceptable scientific standard.

# Reviewer report: Reviewer response for version 2

**DOI:** 10.5256/f1000research.156987.r238722

**Published:** 2024-08-29

**Authors:** Marko Katic

**Affiliations:** 1 University of Zagreb, Zagreb, Croatia

The paper describes performance of several rotary file systems used for root canal preparation. While the presented research is in general sound and interesting, several issues need to be addressed in order to better describe the significance of obtained results:

 1. Better explanation of Z_beta, i.e. why was 80% chosen and how this affects the statistical significance of results.

 2. Preparation of samples - explain how was curvature (10 deg.) determined, which method was used.

 3. In general, using mass to determine the amount of debris has several shortcomings which at least need to be mentioned. This can then be compared to other methods, i.e. Kabil E, et. al. 2021 (ref. 1) and Bago I, et. al. 2021 (ref 2) which are added below.

Is the work clearly and accurately presented and does it cite the current literature?

Yes

If applicable, is the statistical analysis and its interpretation appropriate?

Partly

Are all the source data underlying the results available to ensure full reproducibility?

Yes

Is the study design appropriate and is the work technically sound?

Partly

Are the conclusions drawn adequately supported by the results?

Partly

Are sufficient details of methods and analysis provided to allow replication by others?

Yes

Reviewer Expertise:

Metrology, especially dimensional measurements with interdisciplanry applications.

I confirm that I have read this submission and believe that I have an appropriate level of expertise to confirm that it is of an acceptable scientific standard, however I have significant reservations, as outlined above.

## References

[1] : Micro-computed Evaluation of Canal Transportation and Centering Ability of 5 Rotary and Reciprocating Systems with Different Metallurgical Properties and Surface Treatments in Curved Root Canals. *J Endod* .2021;47(3) : 10.1016/j.joen.2020.11.003 477-484 10.1016/j.joen.2020.11.003 33217469

[2] : Effect of a novel laser-initiated photoacoustic activation of a solvent or sodium hypochlorite in the removal of filling remnants after retreatment of curved root canals. *Photodiagnosis Photodyn Ther* .2021;36: 10.1016/j.pdpdt.2021.102535 102535 10.1016/j.pdpdt.2021.102535 34536606

# Reviewer report: Reviewer response for version 2

**DOI:** 10.5256/f1000research.156987.r302255

**Published:** 2024-07-26

**Authors:** Sandeep Dubey

**Affiliations:** 1 CONSERVATIVE DENTISTRY & ENDODONTICS, Babu Banarasi Das College of Dental Sciences, Lucknow, UTTAR PRADESH, India

- Apical extrusion depends on the cross-section of the rotary files as also mentioned in the  this study. Both ProTaper Next and 2 Shape have different cross-sections, yet both demonstrate a similar amount of apical extrusion in this study. The authors should discuss the possible causes in the manuscript.
- None of the tables were cited inside the text. Please cite them in proper locations.
- Please add a statement regarding acceptance or rejection of the null hypothesis in the first paragraph of the discussion.

Is the work clearly and accurately presented and does it cite the current literature?

Partly

If applicable, is the statistical analysis and its interpretation appropriate?

Yes

Are all the source data underlying the results available to ensure full reproducibility?

Yes

Is the study design appropriate and is the work technically sound?

Yes

Are the conclusions drawn adequately supported by the results?

Yes

Are sufficient details of methods and analysis provided to allow replication by others?

Yes

Reviewer Expertise:

Dentistry, Endodontics, Operative Dentistry, Dental Material

I confirm that I have read this submission and believe that I have an appropriate level of expertise to confirm that it is of an acceptable scientific standard, however I have significant reservations, as outlined above.

# Reviewer report: Reviewer response for version 2

**DOI:** 10.5256/f1000research.156987.r212695

**Published:** 2023-10-16

**Authors:** Sekar Mahalaxmi

**Affiliations:** 1 SRM Dental College, Chennai, Tamil Nadu, India

The authors have done the corrections satisfactorily. However, English language check needs to be done once, before indexing.

Is the work clearly and accurately presented and does it cite the current literature?

Partly

If applicable, is the statistical analysis and its interpretation appropriate?

Yes

Are all the source data underlying the results available to ensure full reproducibility?

Yes

Is the study design appropriate and is the work technically sound?

Yes

Are the conclusions drawn adequately supported by the results?

Yes

Are sufficient details of methods and analysis provided to allow replication by others?

Yes

Reviewer Expertise:

Endodontics, pulp-dentin regeneration, nanotechnology, remineralisation, bonding

I confirm that I have read this submission and believe that I have an appropriate level of expertise to confirm that it is of an acceptable scientific standard.

# Reviewer report: Reviewer response for version 1

**DOI:** 10.5256/f1000research.148345.r194274

**Published:** 2023-08-24

**Authors:** Sekar Mahalaxmi

**Affiliations:** 1 SRM Dental College, Chennai, Tamil Nadu, India

**Abstract:**

 ‘A total of 60 freshly extracted single‐rooted mandibular premolars were use.’ It should be ‘used’.

**Introduction:**

 Very well written introduction. A few comments though:

- Para 2: The authors can avoid old references and refer to some of the latest articles.
- Para 5: ‘After glide path preparation, the Prime (26/4%) file is recommended to use directly. The Small file is only used when the Prime file fails to advance smoothly or when the dentist is unsure about the Prime file. Through its slim design, regressive taper, instrument geometry and heat treatment, TruNatomy files conserve tooth integrity while preserving maximum pericervical dentine.’ This segment is unnecessary since the study involves evaluation of apical debris extrusion.
- Paras 5, 6 and 7: Instead of providing the manufacturers’ details of the file system, the authors can state some studies comparing these instruments in terms of apical debris extrusion and other relevant parameters.
- What is the rationale of this study? Lacunae in literature is missing.
- In the aim the authors have mentioned ‘….and evaluate the effectiveness of their design in minimizing the amount of apical debris extrusion during use.’ How was this evaluated?

**Methodology:**

- Sample size calculation: sample size has been calculated for 2 groups while the study has 3 groups?
- ‘The irrigation was supposed to be passive irrigation as much as possible.’ This statement is very ambiguous and unscientific, please modify and mention the exact method of irrigation.

**Results:**

- Tables 1 and 2 can be avoided and table 3 can contain mean values with SD with comparisons between groups.
- 'P-value' should be 'p-value'.

**Discussion:**

- ‘A primary objective of cleaning and shaping involves preventing apical extrusion of debris and irrigants into the periradicular tissues.’ Please rephrase as the primary objective of C & S is not preventing debris extrusion.
- The rationale of testing the mentioned three instruments among the plethora of rotary instruments available, is inadequate. Just choosing three instruments randomly does not contribute to comparison.
- Previous literature on debris extrusion with contradictory results needs to be discussed as to the variation in the results obtained.
- Authors' names can be mentioned without the year (in 2018, in 2019, etc. can be deleted).
- Discussion needs some rearrangements in terms of explanation for debris extrusion. A bit confusing, when relating to the different factors causing apical extrusion of debris.

Is the work clearly and accurately presented and does it cite the current literature?

Partly

If applicable, is the statistical analysis and its interpretation appropriate?

Yes

Are all the source data underlying the results available to ensure full reproducibility?

Yes

Is the study design appropriate and is the work technically sound?

Yes

Are the conclusions drawn adequately supported by the results?

Yes

Are sufficient details of methods and analysis provided to allow replication by others?

Yes

Reviewer Expertise:

Endodontics, pulp-dentin regeneration, nanotechnology, remineralisation, bonding

I confirm that I have read this submission and believe that I have an appropriate level of expertise to confirm that it is of an acceptable scientific standard, however I have significant reservations, as outlined above.

# Response

**Published:** 2023-10-02

**Authors:** Rutuja Rajnekar

**Affiliations:** dentistry, datta meghe institute of higher medical research, wardha, maharashtra, India

- Changes done in the abstract.
- Changes done in the introduction section.
- Stated the rationale & and stated the method of evaluation.
- Correction done in methodology.
- Results corrected.
- Discussion corrected.
- Rephrasing done with the C & S statement.
- Rationale mentioned.
- Years removed​​​​​​.
